# Clearance of amyloid β-protein and its role in the spreading of Alzheimer's disease pathology

**DOI:** 10.3389/fnagi.2015.00025

**Published:** 2015-03-09

**Authors:** Dietmar R. Thal

**Affiliations:** Laboratory of Neuropathology – Institute of Pathology, Center for Biomedical Research, University of UlmUlm, Germany

**Keywords:** amyloid β-protein, tau-protein, perivascular drainage, propagation of protein aggregates, cellular clearance

Amyloid β-protein (Aβ) containing amyloid plaques and abnormal phosphorylated τ-protein containing neurofibrillary tangles (NFTs) are hallmark lesions of Alzheimer's disease. Both Aβ plaques and NFTs show hierarchical patterns in which the areas of the brain are subsequently affected by Aβ plaques and NFTs, respectively (Braak and Braak, 1991; Thal et al., 2002). Aβ plaques start to develop in the neocortex (phase 1) and spread from there into allocortical regions (phase 2), diencephalon, basal forebrain and striatum (phase 3), midbrain and medulla oblongata (phase 4), and finally into the pons and the cerebellum (phase 5) (Thal et al., 2002). The first NFTs in the brain hemispheres are found in the transentorhinal cortex (stage I), then in the entorhinal cortex (stage II), the hippocampus (stage III), the temporal cortex (stage IV), further neocortical areas except the primary fields (stage V), and, finally, also in primary cortical areas, such as the primary visual cortex (stage VI) (Braak and Braak, 1991). Axonal connections between subsequently affected brain regions suggest that AD pathology spreads along neuronal pathways (Thal et al., 2002; Braak and Del Tredici, 2011).

Insufficient clearance of Aβ has been considered to play an essential role in the pathogenesis of AD. Clearance mechanisms that contribute to Aβ elimination from brain are cellular enzymatic proteolysis in glial cells, neurons or in the extracellular space (Qiu et al., 1998; Yamaguchi et al., 1998; Iwata et al., 2000; Thal et al., 2000; Farris et al., 2003), transport through the blood-brain barrier (Shibata et al., 2000; Ito et al., 2007), and perivascular drainage (Weller et al., 2008) (Figure 1A).

**Figure 1:**
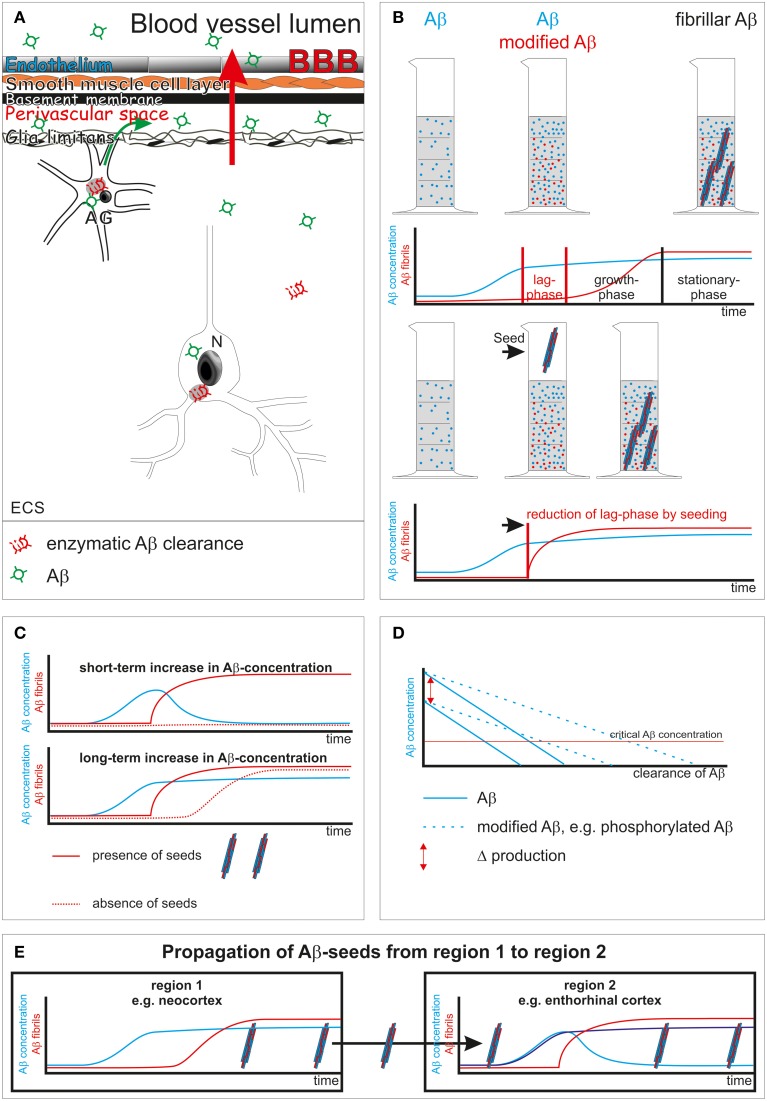
**Schematic representation of Aβ clearance and propagation. (A)** Aβ clearance mechanisms: enzymatic clearance within neurons (N) and glial cells [here shown in the example is an astrocyte (AG)] or in the extracellular space (ECS) (Qiu et al., 1998; Yamaguchi et al., 1998; Iwata et al., 2000; Thal et al., 2000; Farris et al., 2003); transport through the blood-brain barrier (BBB) into the blood (red arrow) (Shibata et al., 2000; Ito et al., 2007); drainage into the perivascular space (green arrow) (Weller et al., 2008). **(B)** Aβ aggregation into fibrils shows a lag-phase that is reduced by the presence of seeds (Thal et al., 2015). **(C)** Initiation of Aβ fibril formation in the presence of seeds starts immediately after a critical concentration is passed even for a short period of time. In the absence of seeds a longer increase of Aβ concentration may be necessary to pass the lag-phase for initiating Aβ aggregation. **(D)** Influence of clearance rate, Aβ-production and its composition [presence of modified forms of Aβ that are cleared less effectively than normal Aβ (Russo et al., 2002; Kumar et al., 2012)] on a potentially critical concentration of Aβ for disease progression. N-terminal truncated, pyroglutamate-modified Aβ is less soluble than non-modified full-length Aβ (Schlenzig et al., 2009). In other words, this modification of Aβ modified its critical concentration for aggregation. **(E)** Propagation of AD pathology, e.g., Aβ plaques, takes place when seeds of Aβ are transported to a second primarily non-involved brain region. As soon as the concentration of Aβ passes a critical level Aβ aggregation and fibril formation takes place, leading to the deposition of Aβ plaques in the secondarily affected brain region.

Here, I will discuss the potential impact of impaired Aβ clearance on propagation mechanisms for Aβ and τ.

## Biophysical and biochemical prerequisites for Aβ and τ protein aggregation

Biophysically, protein aggregation takes place once a critical concentration of proteins has been passed. Fibril formation sets in after a concentration-dependent lag-phase, i.e., the time interval between passing a critical concentration and forming fibrils (Figure 1B) (Chirita et al., 2005). Within the lag-phase, assembly of proteins into non-fibrillar intermediates, i.e., oligomers of all sizes, precedes fibril formation (Thal et al., 2015). Proteins in general differ in their capability to aggregate, and their assembly can be further modulated by chaperones (Gething and Sambrook, 1992). Posttranslational modifications of proteins, such as N-terminal truncation and pyroglutamate formation, phosphorylation, or glycation increase the tendency of Aβ or τ to form aggregates (Necula and Kuret, 2004; Schlenzig et al., 2009; Kumar et al., 2011). Seeds of preaggregated fibrils can reduce the lag-phase dramatically and trigger immediate aggregation (Figure 1B).

In other words, a prerequisite for Aβ and/or τ protein aggregation is either a sufficient concentration of Aβ or τ over a period of time long enough to pass the respective lag-phase or the presence of preaggregated seeds to start aggregation as soon as the critical protein concentration has been reached.

## Aβ clearance modulates concentration and onset of fibril formation of Aβ

Sufficient Aβ clearance is required to prevent an increase of the Aβ concentration. In the absence of preaggregated Aβ seeds a short-term increase of Aβ concentration not exceeding the lag-phase may not start the aggregation process. Accordingly, a “long-term” elevation of Aβ concentration is required to initiate the process of Aβ aggregation under normal conditions (Figures 1B,C). As soon as preaggregated Aβ seeds are present aggregation of Aβ will be initiated once the critical Aβ concentration has been passed as demonstrated in animal models for Aβ deposition (Meyer-Luehmann et al., 2006) (Figure 1C).

## Impairment of Aβ clearance by modified forms and/or Aβ intermediates

Posttranslationally modified forms of Aβ are cleared less efficiently from the brain as shown for N-terminal truncated and pyroglutamate-modified Aβ and phosphorylated Aβ (Russo et al., 2002; Kumar et al., 2012) (Figure 1D). Oligomeric Aβ intermediates are stable and quite resistant to degeneration (Viola and Klein, 2015). Moreover, Aβ oligomers alter proteasomal clearance (Cecarini et al., 2012). Accordingly, it is tempting to speculate that even low amounts of Aβ intermediates and fibrils as well as posttranslational modifications of Aβ foster disease progression not only by acting as seeds but also by their property of impairing physiological Aβ clearance and, thereby, increasing Aβ concentration.

## Spreading of Aβ pathology

Spreading of Aβ pathology means that Aβ aggregation and deposition already took place at least in the neocortex and a second region becomes involved in this process. Preaggregated neocortical Aβ may be transported into secondarily affected brain regions by glial cells or neurons or by diffusion (Guo and Lee, 2014; Thal et al., 2015). In the event that seeds prevail in a given brain region Aβ aggregation will be initiated as soon as a critical concentration has been passed (Figure 1E). Given the prevalence of cortical Aβ plaques (i.e., aggregated Aβ) in most elderly individuals (Braak et al., 2011) it is tempting to speculate that progression of Aβ pathology into further brain regions can be triggered by insufficient local clearance and subsequently increased Aβ levels and by seeding its aggregation. If so, continuously lowering Aβ concentration to avoid even short-term increases might be effective in preventing propagation of Aβ pathology, similar to the inactivation of Aβ seeds as shown for continuous Aβ antibody treatment in Aβ-producing mice (Paganetti et al., 2013).

## Aβ and τ

Animal experiments in τ-transgenic mice have demonstrated that injecting Aβ or crossbreeding these animals with Aβ-producing amyloid precursor protein transgenic mice accelerates and increases τ-pathology (Gotz et al., 2001; Lewis et al., 2001). Moreover, anti-Aβ antibody treatment reduced τ-pathology in an Aβ and τ-pathology producing mouse model (Oddo et al., 2004). As such, it is tempting to speculate that there is cross-seeding of τ-pathology by Aβ aggregates *in vivo* similar as *in vitro* (Lasagna-Reeves et al., 2010). Arguments against relevant cross-seeding of Aβ and τ in the AD brain may be (a) that τ aggregates are intracellular aggregates while Aβ plaques are extracellular protein aggregates, and (b) Aβ plaques develop first in the neocortex whereas NFTs are found in this part of the brain only in advanced stages of AD. However, Aβ also occurs intracellularly (Gouras et al., 2000) and τ aggregates are transported through the extracellular space (Kfoury et al., 2012), indicating that an interaction of τ and Aβ may be possible either intra- or extracellularly. Moreover, as discussed for Aβ, a sufficient protein concentration is essential for the initiation of protein aggregation. Accordingly, cross-seeding of τ by Aβ cannot take place if there is not enough aggregation-prone τ protein even in the presence of huge amounts of Aβ seeds. Such a constellation may apply for neocortical brain regions in early stages of AD. Therefore, it seems to be likely that cross-seeding of Aβ and τ contributes to the development of AD and may be modulated by changing the clearance of Aβ. Cross-seeding of Aβ and τ does not exclude the independent aggregation of τ, as τ-aggregates have been shown to trigger the initiation of τ-pathology (Clavaguera et al., 2009).

## Conclusion

The hypothesis that insufficient Aβ clearance contributes to the development of AD does not contradict a major role of preaggregated Aβ and/or τ seeds in the propagation of the disease. Moreover, improving Aβ clearance, e.g., by enhancing its enzymatic degradation or vaccination strategies, may be capable of slowing down disease propagation given the relevance of Aβ as a substrate for the protein aggregation process in AD.

### Conflict of interest statement

The author discloses the following potential conflicts of interest: DRT received consultancies from Covance Laboratories (UK) and GE-Healthcare (UK), a speaker honorarium from GE-Healthcare (UK) and collaborated with Novartis Pharma Basel (Switzerland).
